# Supplementary material for: Subnational variations in the quality of household survey data in sub-Saharan Africa
Source: Nat Commun. 2025 Apr 22;16:3771. doi: 10.1038/s41467-025-58776-5 (PMC12015360; doi:10.1038/s41467-025-58776-5)
Supplement: Supplementary file 1 — Supplementary Information [file 41467_2025_58776_MOESM1_ESM.pdf]

Supplementary Information

Subnational variations in the quality of household survey data in sub-Saharan Africa

Valentin Seidler[1*], Edson C. Utazi[2], Amelia B. Finaret[3,6], Sebastian Luckeneder[4], Gregor Zens[5] , Maksym Bodarenko[2], Abigail W. Smith[6], Sarah E.K. Bradley[7], Andrew J. Tatem[2], Patrick Webb[8]

[1]Central European University; Vienna, Austria; [2]Worldpop, University of Southampton, Southampton, UK; [3]University of Edinburgh, Global Academy of Agriculture and Food Systems, Edinburgh, Scotland, UK; [4]Vienna University of Economics and Business, Department of Socioeconomics, Vienna, Austria; [5]International Institute for Applied Systems Analysis (IIASA), Laxenburg, Austria; [6]Allegheny College, Department of Global Health Studies, Meadville, Pennsylvania, USA; [7]Independent Public Health Demographer, Washington D.C., USA; [8]Tufts University, Friedman School of Nutrition Science and Policy, Boston, Massachusetts, USA.

Corresponding author information: *Valentin Seidler (email: seidlerv@ceu.edu)

List of Supplementary Items

Supplementary Figure 1: Example of spherical mesh for West Africa 7

Supplementary Figure 2 Minimum, maximum and average (population weighted) value of data errors across Demographic and Health Survey (DHS) clusters, summarized by country 8

Supplementary Figure 3 Correlations between data quality indicators 9

Supplementary Figure 4: Predicted data quality by distance to closest Digital Number (DN) 15 nighttime light emitting source in km (logarithmic scale) along regions in sub-Saharan Africa. 10

Supplementary Figure 5: Predicted data quality by distance to closest Digital Number (DN) 10 nighttime light emitting source in km (logarithmic scale) 11

Supplementary Figure 6: Predicted data quality by distance to closest Digital Number (DN) 20 nighttime light emitting source in km (logarithmic scale) 12

Supplementary Figure 7: Predicted data quality by distance to closest settlement in km (logarithmic scale) 13

Supplementary Figure 8: Predicted data quality by distance to closest urban agglomeration of at least 10,000 inhabitants in km (logarithmic scale). 14

Supplementary Figure 9: Estimated coefficients of distance to the closest Digital Number (DN) 15 nightlight pixel for Demographic and Health Survey (DHS) survey rounds between 2006 and 2022. 15

Supplementary Figure 10: Estimated coefficients of distance to the closest settlement for Demographic and Health Survey (DHS) survey rounds between 2006 and 2022 16

Supplementary Figure 11: Estimated coefficients of distance to the closest urban agglomeration of at least 10,000 inhabitants for Demographic and Health Survey (DHS) survey rounds between 2006 and 2022.....17

Supplementary Figure 12: Associations between estimated coefficients of distance to the closest Digital Number (DN) 15 nightlight pixels and the average value of data quality indicators. 18

Supplementary Figure 13: Associations between estimated coefficients of distance to the closest settlement and the average value of data quality indicators 19

Supplementary Figure 14: Associations between estimated coefficients of distance to the closest urban agglomeration of at least 10,000 inhabitants and the average value of data quality indicators 20

Supplementary Figure 15: Correlations between a) the sampling uncertainty of ’contraceptive use’ with ’incomplete age’ and b) of ’stunted children’ with ’flagged height-for-age (HAZ)'. 21

Supplementary Figure 16: Modelling regions in sub-Saharan Africa. 22

Supplementary Figure 17: Sample plots of the covariates against the empirical logit transform of age heaping across four African regions 23

Supplementary Figure 18: Plots of variograms of residuals from non-spatial models fitted using each of the modelled indicators. 24

Supplementary Table 1: Sources for covariates and auxiliary data 25
Supplementary Table 2: Summary statistics for the covariates 26
Supplementary Table 3: Results of k-fold cross-validation exercise to assess the predictive performance of the fitted models for each African region...27
Supplementary Table 4: Parameter estimates for age heaping 28
Supplementary Table 5: Parameter estimates for flagged height-for-age (HAZ) 29
Supplementary Table 6: Parameter estimates for stunting prevalence ('stunted children') 30
Supplementary Table 7: Parameter estimates for contraceptive use. 31
Supplementary Table 8: Parameter estimates for incomplete age 32
Supplementary Table 9: Completed Demographic and Health Survey (DHS) rounds and selected datasets.........33
Supplementary Table 10: Spatial autocorrelation for 'age heaping' at the district level. 34
Supplementary Table 11: Spatial autocorrelation for 'flagged height-for-age (HAZ)' at the district level. 35
Supplementary Table 12: Spatial autocorrelation for 'incomplete age' at the district level..................................36

Supplementary Note 1: Selected relevant literature by development and agricultural economists

Development economists have long emphasized the importance of reliable demographic and income-related data in Africa[1,2]. More recent studies by development economists have used micro-level surveys in Sub-Saharan Africa to examine measurement errors in agricultural surveys[3–5]. Several studies have investigated measurement errors related to self-reported data and their impact on agricultural measures, such as agricultural input, production data, and land area measurement[3,4,6–8]. Possible explanations for errors that have been identified in the literature include recall bias, underreporting, respondents' propensity to round numbers, cultural factors, logistics of survey implementation, and overreporting[7,9–11]. Development economists have proposed methods to improve data quality, such as utilizing geocoded data for measuring land area[4,6] or creating subnational indices of spatial fragility[12] and local vulnerability[13].

Supplementary Note 2: Data quality indicators

We focused on data quality indicators of age and anthropometric measurements of young children and their mothers, because these indicators are widely used in the literature and among practitioners and because they have particularly important roles in health and demographic research.

Incomplete age - the share of interviewed women aged 15 – 49 years with less than month/year precision (e.g., year only, or no response at all), relative to the population in the cluster.

Background information: Date of birth is particularly difficult to record in national household surveys when birth certificates are missing. Remembering exact birthdate and age estimates based on local events calendar are time consuming and provide less precise information. Assessing the completeness of age data is therefore an important aspect of verifying survey data quality.

Relevance for assessing data quality: Between 2010 and 2019, only 39% of births were registered in Eastern and Southern Africa and 53% in West and Central Africa.[14] Measurement error and missing data on ages affect sample selection, because of inclusion criteria for the survey itself and for individual questions in the survey may be based on age, and age-related measurement error is not likely to be just random.[15]

Age heaping - the proportion of reported ages ending in 5 or 0 of all adults between the ages of 23 and 62.

Background information: Age heaping (also known as digit preference) occurs when reported ages do not follow a uniform distribution across all possible numerical digits from 0-9 as it should be expected for large enough groups of people. A population may have heaped ages when birthdates are uncertain and best guesses are being used or rounding to the nearest round age (ending most commonly in 5 or 0) by respondents or enumerators. For survey respondents, heaping is very common in wide range of settings, such as responding to questions about the number of times a certain place was visited, or the number of servings of fruits and vegetables that were consumed in the previous day. Age heaping may be intentional or un-intentional. Heaped ages may indicate

a lack of care of enumerators. It is often the result of enumerators' fatigue or of respondents not remembering their age.[16–18]

Whipple's Index: Among the possible approaches of assessing errors in reported ages, we choose a method close to the Whipple's Index, which is the most intuitive approach for a general audience. Among all reported ages of adults, ages ending in 5 or 0 should naturally occur in 20% of reported ages. A value above 20% indicates age heaping.

Relevance for assessing data quality: Age heaping is a commonly understood indicator of general data quality, not just for accurate information on age itself. It draws in doubt the accuracy of other variables, which depend on age to be calculated.[19] There are analytical strategies to address age heaping, such as imputation, which may require additional data.

Flagged HAZ - the share of biologically implausible or missing values for the attained height-for-age z-scores of children under five according to World Health Organization (WHO) standards.

Background information: Nutritional status is indicated in part by height-for-age (HAZ), for which deficits indicate possible linear growth faltering, a condition associated with reduced cognitive capacity, reduced educational attainment, reduced earnings, and increased morbidity and mortality.[20] Heights and weights are comparably easier to measure in many contexts than biochemical, clinical, or dietary signs of nutritional status, but the measurement of child linear growth still requires accurate information on date of birth sex, and length or height. Measurement errors occur because infants, toddlers, and preschool aged children are difficult to measure despite robust enumerator training and preparation. For example, even random errors and artefacts in recording the month of birth of children can produce nonrandom pattern in in HAZ[15]. Normal child behaviors may interfere with accurate measurement. Children who are feeling sick or unwell may not be able to have an accurate measurement or any measurement at all. Some respondents may not want to have their children measured and may decline.

Flagging system according to WHO standards: Implausible values are defined as z-scores that fall outside a specified range of values which are considered biologically possible. The currently recommended flagging system to detect implausible z-score values was defined in 2006 on the release of the WHO Child Growth Standards. The WHO flagging system is not without dispute in the literature. They define rather broad flagging standards and likely do not detect all values due to measurement errors, if they fall within the plausible range. The share of implausible z-score values is calculated using unweighted sample weights for all children measured in the entire sample. For the currently recommended fixed exclusions approach, -6 to +6 HAZ z-score values outside the following intervals are considered implausible.[21]

Relevance for assessing data quality: Flagged HAZ and other flagged anthropometric indicators are widely used measures for the overall survey data quality. Owing to the broad range of HAZ values considered plausible according to WHO, a high percentage of flagged values reliably indicates poor data quality, however a low percentage does not necessarily imply adequate data quality since values that are inaccurate may still occur within the WHO flag range.

Supplementary Note 3: Limitations

Both the data used in the analysis and the methods are subject to several limitations. Spatial data gaps between DHS survey locations may introduce varying degrees of uncertainty in our estimates. Estimation uncertainty may vary within and across countries. These considerations

also apply – to a lesser extent – to the aggregated analyses on the sub-national and are relevant to any geolocated household-survey based data set. We expect that statistical uncertainty is lowest where it matters most, namely in highly populated areas where most survey locations are located. We explicitly show and discuss sampling-based uncertainty in the context of Figure 3. Second, DHS survey locations are randomly displaced in space for data confidentiality reasons, further increasing the level of spatial uncertainty. However, this is not expected to influence the overall findings of this paper significantly, as most of our discussion takes place on a more aggregated spatial scale that goes beyond five kilometers, which is the maximum distance for survey location displacement in the DHS methodology for 99% of clusters[22]. However, practitioners should keep this in mind when interpreting results on a very fine level of spatial granularity. In addition, the covariate layers we use to extract external information for each DHS cluster do not vary substantially at this high resolution. Hence, we expect that using, e.g., buffering extraction surrounding GPS locations instead of point extraction using GPS locations will give extremely similar results and that our overall findings will remain essentially unchanged.

Our model uses four covariates. Including more covariates would not challenge our argument, but make the paper a more technical exercise which we found less suited for a general audience that should be informed about the magnitude of the problem. We hope that subsequent studies will replicate or challenge our findings with more sophisticated models. Third, in terms of statistical methodology, our modeling framework is, like any statistical model, an abstraction of reality and involves several assumptions and simplifications. However, the predictive framework we build is state-of-the-art in the contemporaneous literature on small-scale mapping exercises. In addition, we conduct extensive model validation exercises to ensure a good fit and high quality of the obtained predictions. Finally, and with exception of the analyses behind Supplementary Supplementary Figure 9-11, our dataset is based on a cross-country sample of surveys. Hence, the country-level data quality differences we document could either be due to actual underlying country-level factors or reflect differences across survey rounds. As researchers and practitioners preferably use the most recent survey available for each country, this does not to change the major arguments of the article which are to caution the unchecked analytical use and the interpretation of household survey data in research and to potentially contribute to efforts to improve future survey rounds.

Supplementary Figures

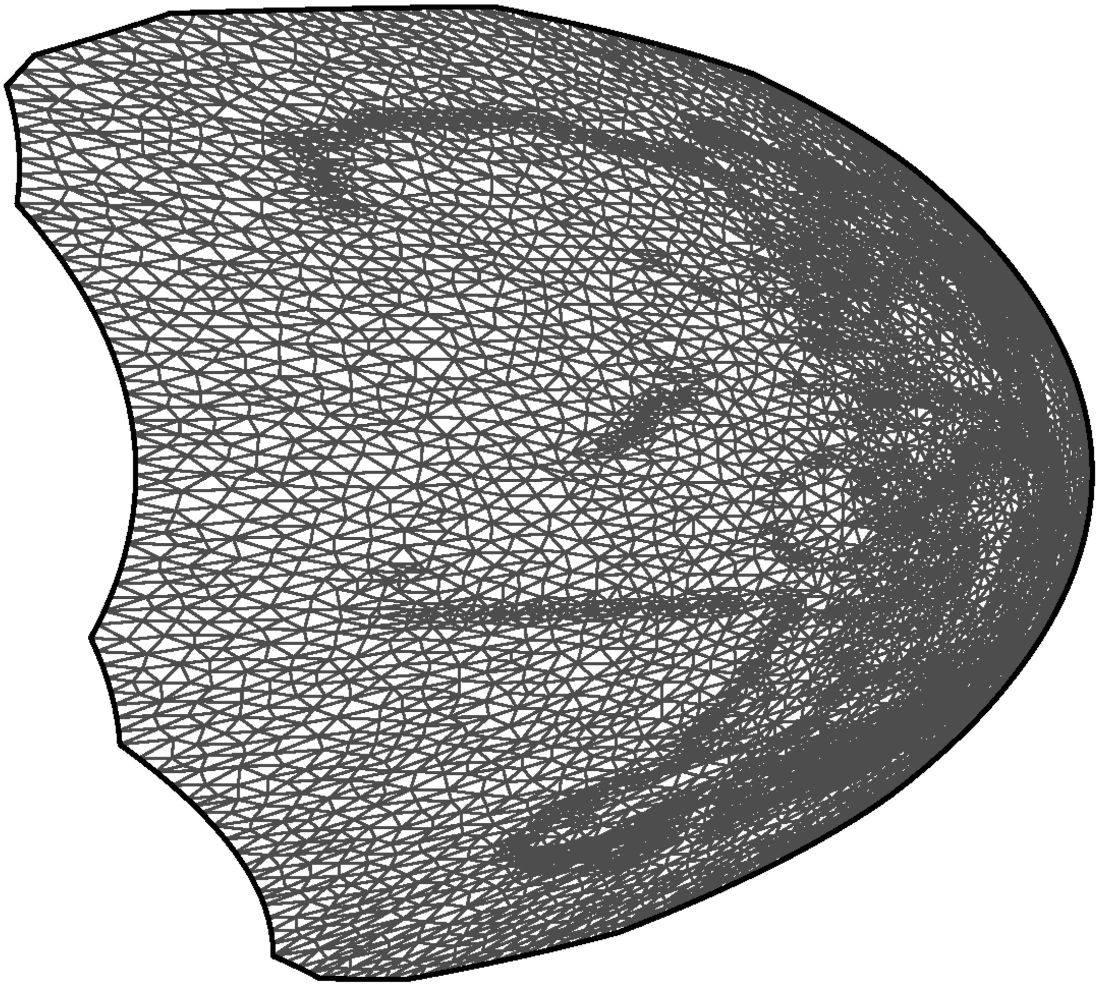

Supplementary Figure 1: Example of spherical mesh for West Africa. Our spatial model, based on the integrated nested Laplace approximation (INLA) and the stochastic partial differential equation (SPDE), specifies a fine triangulation mesh to approximate the spatial random effect for each African region.

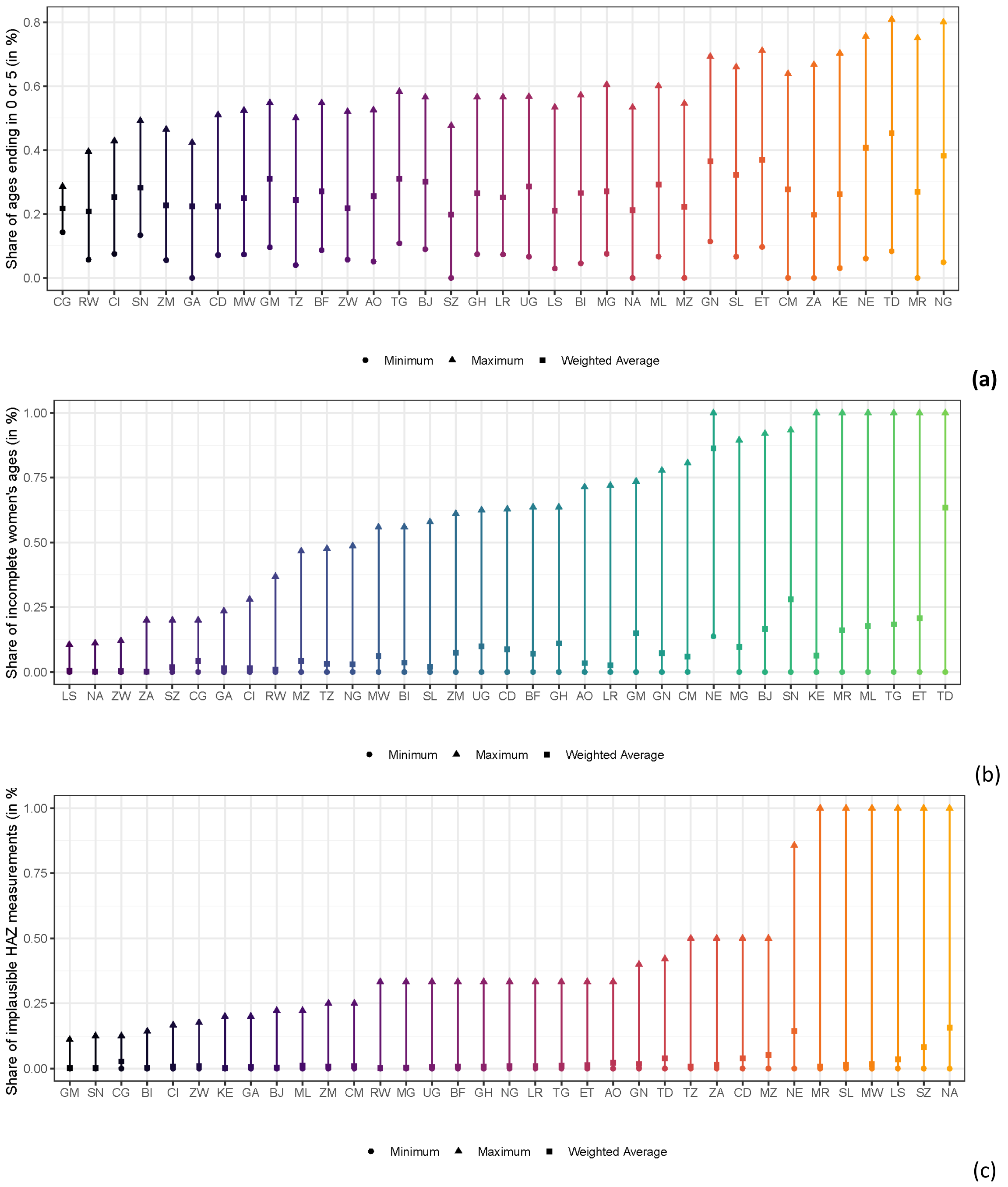

Supplementary Figure 2: Minimum, maximum and average (population weighted) value of data errors across Demographic and Health Survey (DHS) clusters, summarized by country (a) share of reported ages ending in 5 or 0 of all adults between 23 and 62 ('age heaping'), (b) share of interviewed women (15-49 years) with either the year or month of birth reported missing ('incomplete age'), and (c) implausible or missing values for the attained height-for-age z-scores of children under five according to World Health Organization (WHO) standards ('flagged HAZ').

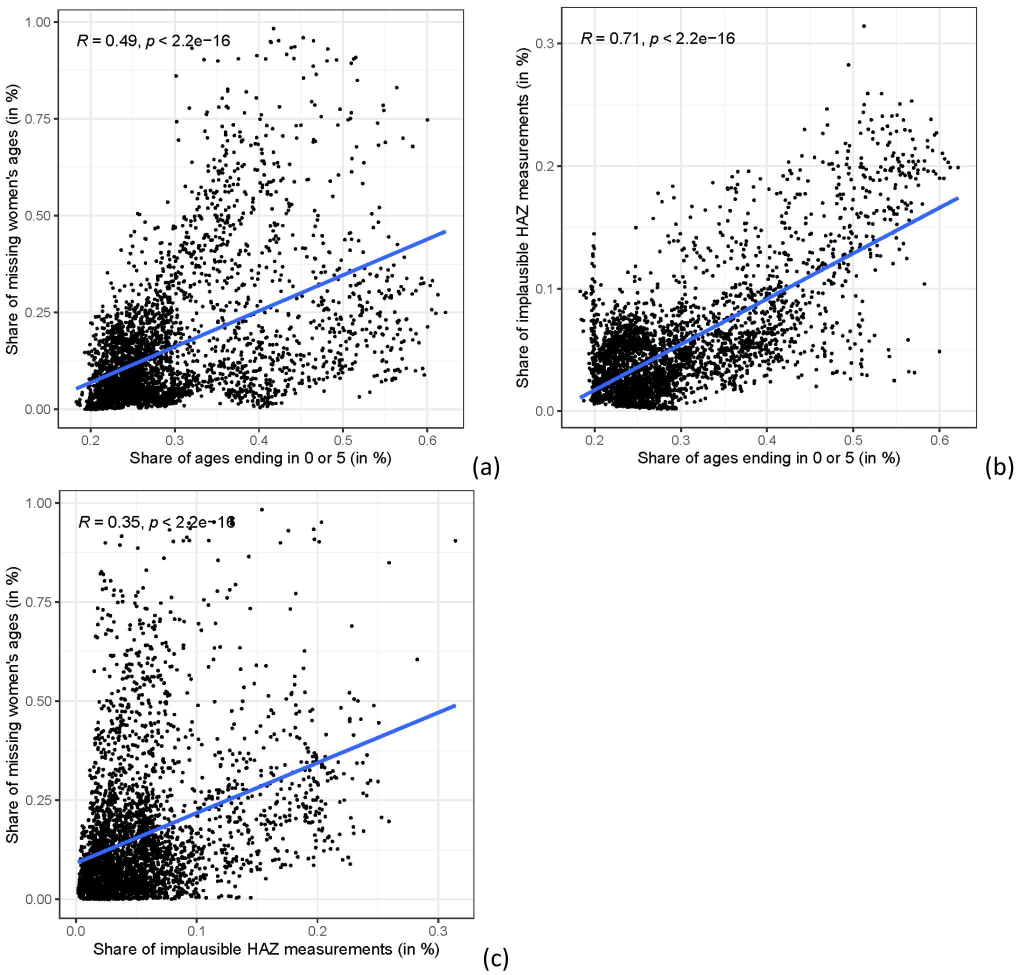

Supplementary Figure 3: Correlations between data quality indicators (a) share of reported ages ending in 5 or 0 of all adults between 23 and 62 ('age heaping') versus share of interviewed women (15-49 years) with either the year or month of birth reported missing ('incomplete age'), (b) share of reported ages ending in 5 or 0 of all adults between 23 and 62 ('age heaping') vs. implausible or missing values for the attained height-for-age z-scores of children under five according to World Health Organization (WHO) standards ('flagged HAZ'), (c) share of implausible or missing values for the attained height-for-age z-scores of children under five according to WHO standards ('flagged HAZ') versus share of interviewed women (15-49 years) with either the year or month of birth reported missing ('incomplete age').

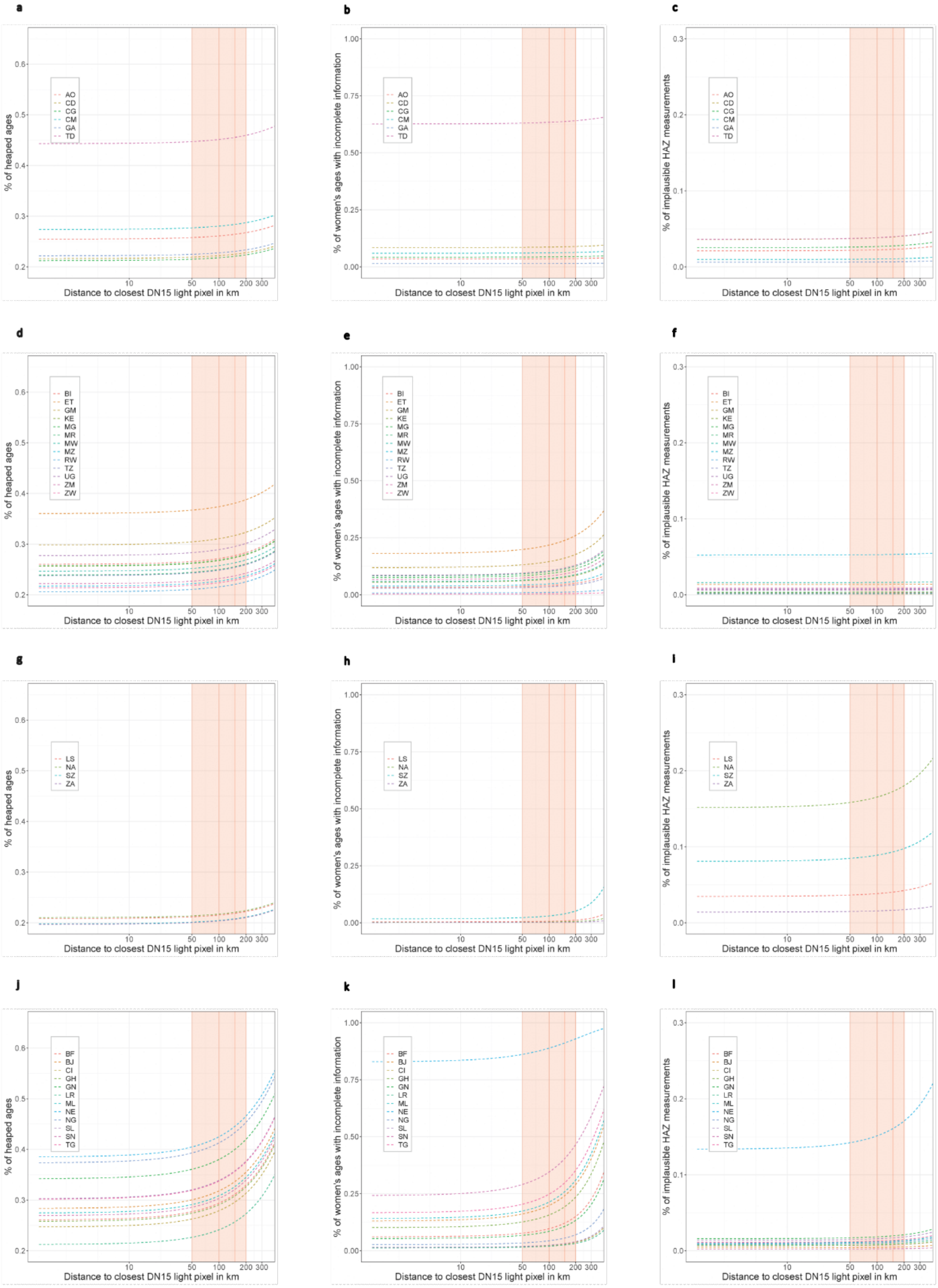

Supplementary Figure 4: Predicted data quality by distance to closest Digital Number (DN) 15 nighttime light emitting source in km (logarithmic scale) along regions in sub-Saharan Africa. a-l, Predictions obtained from regional binomial logistic regressions on distance (in km) to closest DN 15 light pixel of age heaping, incomplete age and flagged height-for-age (HAZ) for the (a-c) central region, (d-f) eastern region, (g-i) southern region, and (j-l) western region. All models include country fixed effects.

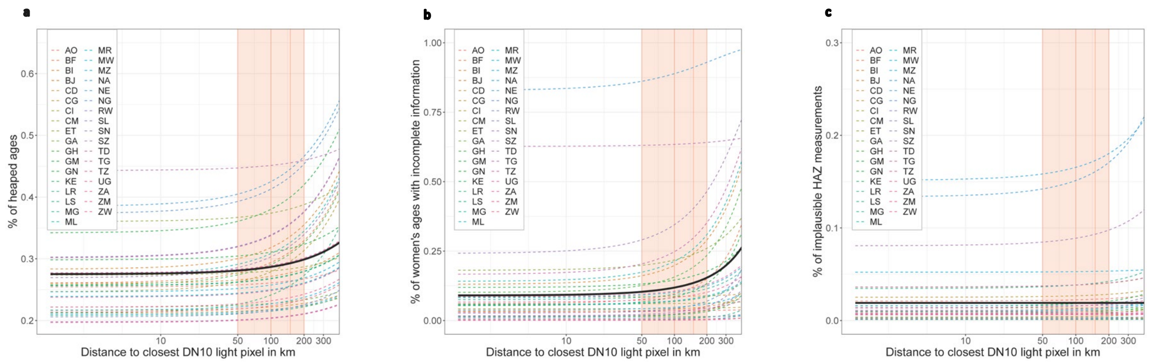

Supplementary Figure 5: Predicted data quality by distance to closest Digital Number (DN) 10 nighttime light emitting source in km (logarithmic scale). a-c, Predictions obtained from regional binomial logistic regressions on distance (in km) to closest DN 10 light pixels of (a) share of reported ages ending in 5 or 0 of all adults between 23 and 62 (’age heaping’), (b) share of interviewed women (15-49 years) with either the year or month of birth reported missing (’incomplete age’), and (c) implausible or missing values for the attained height-for-age z-scores of children under five according to World Health Organization (WHO) standards (’flagged HAZ’). All models include country fixed effects.

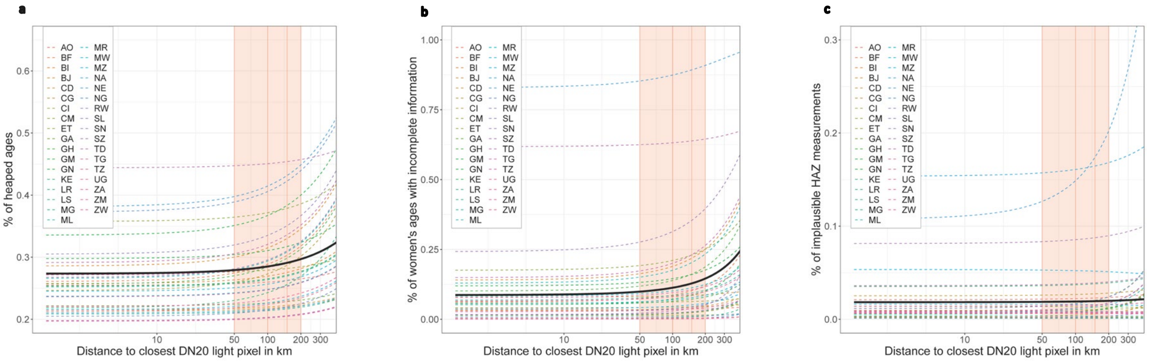

Supplementary Figure 6: Predicted data quality by distance to closest Digital Number (DN) 20 nighttime light emitting source in km (logarithmic scale). a-c, Predictions obtained from regional binomial logistic regressions on distance (in km) to closest DN 20 light pixels of (a) share of reported ages ending in 5 or 0 of all adults between 23 and 62 (’age heaping’), (b) share of interviewed women (15-49 years) with either the year or month of birth reported missing (’incomplete age’), and (c) implausible or missing values for the attained height-for-age z-scores of children under five according to World Health Organization (WHO) standards (’flagged HAZ’). All models include country fixed effects.

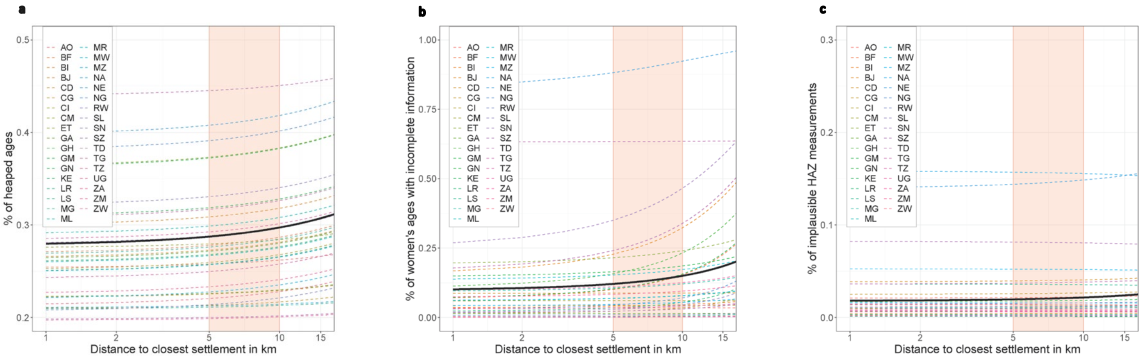

Supplementary Figure 7: Predicted data quality by distance to closest settlement in km (logarithmic scale). a-c, Predictions obtained from regional binomial logistic regressions on distance (in km) to closest settlement of (a) share of reported ages ending in 5 or 0 of all adults between 23 and 62 (’age heaping’), (b) share of interviewed women (15-49 years) with either the year or month of birth reported missing (’incomplete age’), and (c) implausible or missing values for the attained height-for-age z-scores of children under five according to World Health Organization (WHO) standards (’flagged HAZ’). Data by Marconcini et al. All models include country fixed effects.

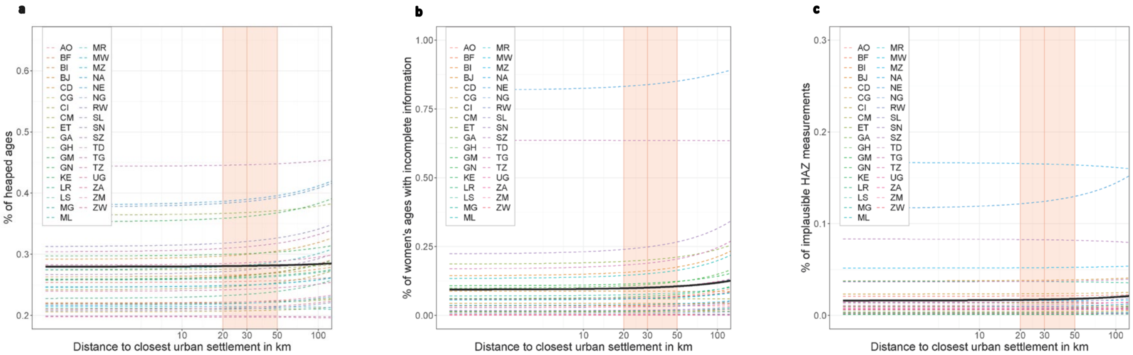

Supplementary Figure 8: Predicted data quality by distance to closest urban agglomeration of at least 10,000 inhabitants in km (logarithmic scale). a-c, Predictions obtained from regional binomial logistic regressions on distance (in km) to closest settlement of (a) share of reported ages ending in 5 or 0 of all adults between 23 and 62 (’age heaping’), (b) share of interviewed women (15-49 years) with either the year or month of birth reported missing (’incomplete age’), and (c) implausible or missing values for the attained height-for-age z-scores of children under five according to World Health Organization (WHO) standards (’flagged HAZ’). Data taken from the OCED Africapolis dataset. All models include country fixed effects.

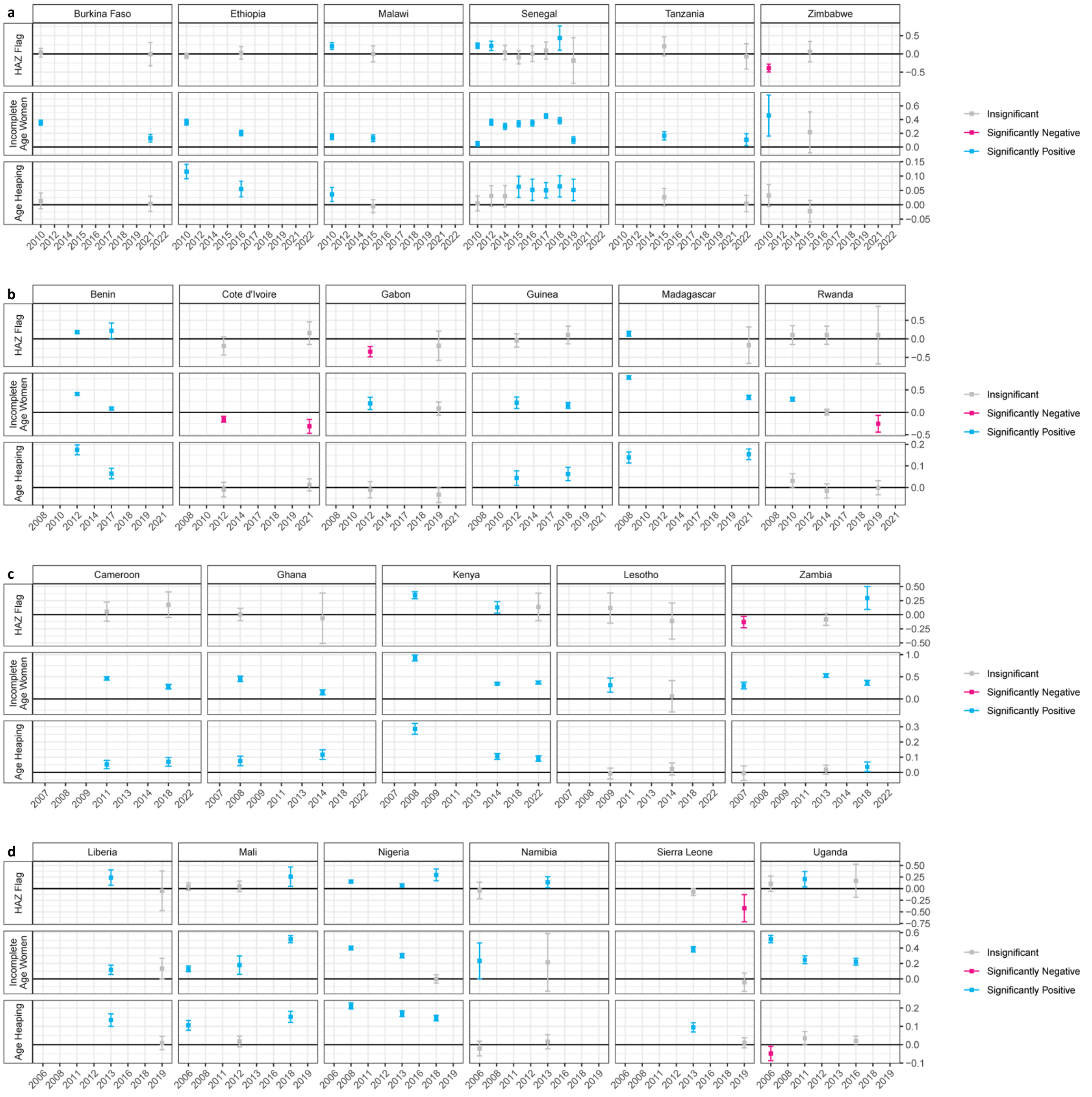

Supplementary Figure 9: Estimated coefficients of distance to the closest Digital Number (DN) 15 nightlight pixel for Demographic and Health Survey (DHS) survey rounds between 2006 and 2022. Coefficients obtained from binomial regressions of the data quality indicators on the distance measure and an intercept term. Regression models have been estimated separately for each distinct survey. Countries with only one survey round were excluded. DHS surveys prior to 2006 were excluded as height-for-age (HAZ) score standards set by World Health Organization (WHO) became effective only in 2006. Error bars correspond to 95% confidence intervals**.**

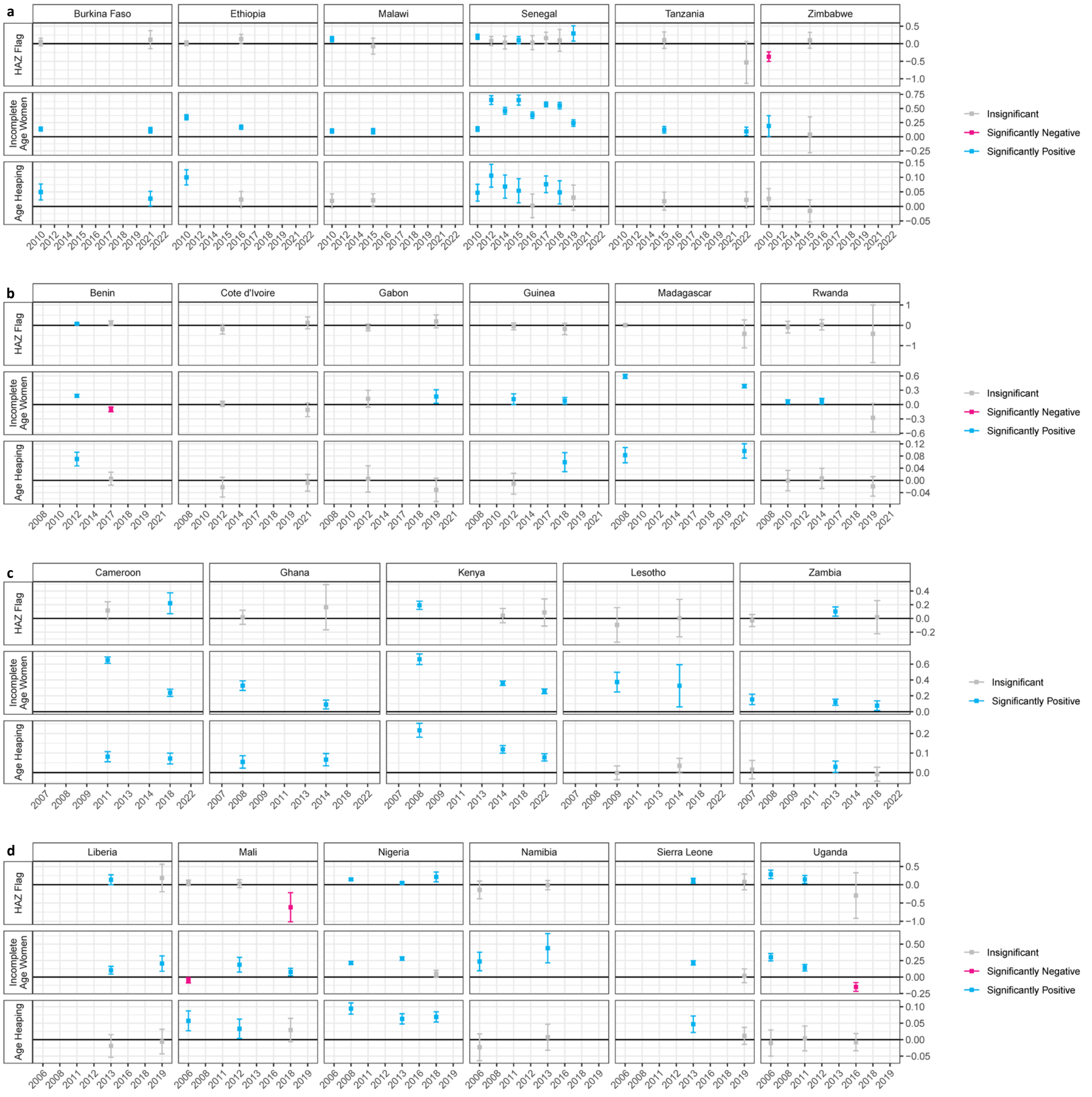

Supplementary Figure 10: Estimated coefficients of distance to the closest settlement for Demographic and Health Survey (DHS) survey rounds between 2006 and 2022. Coefficients obtained from binomial regressions of the data quality indicators on the distance measure and an intercept term. Regression models have been estimated separately for each distinct survey. Countries with only one survey round were excluded. DHS surveys prior to 2006 were excluded as height-for-age (HAZ) score standards set by World Health Organization (WHO) became effective only in 2006. Error bars correspond to 95% confidence intervals.

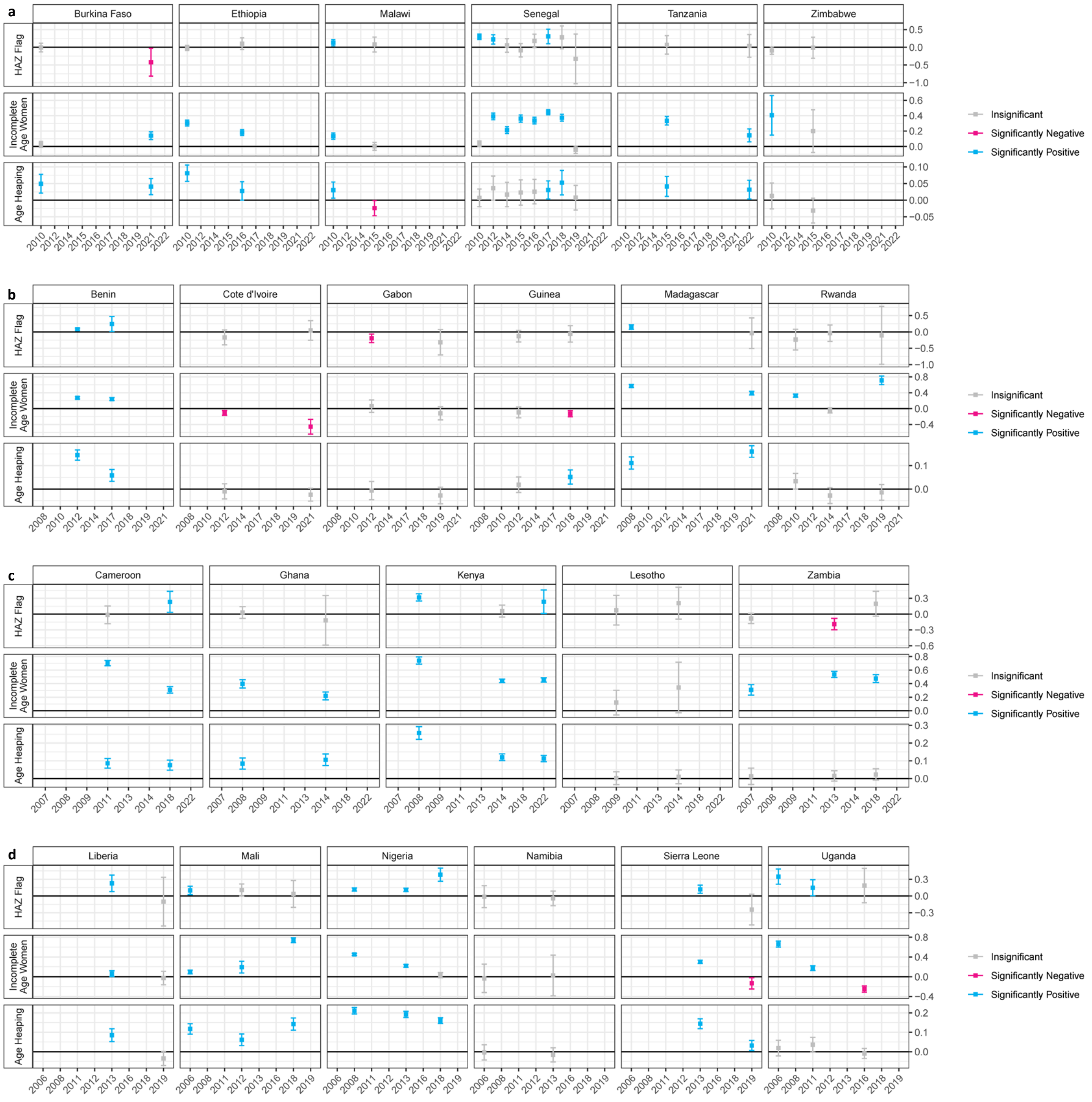

Supplementary Figure 11: Estimated coefficients of distance to the closest urban agglomeration of at least 10,000 inhabitants for Demographic and Health Survey (DHS) survey rounds between 2006 and 2022. Coefficients obtained from binomial regressions of the data quality indicators on the distance measure and an intercept term. Regression models have been estimated separately for each distinct survey. Countries with only one survey round were excluded. DHS surveys prior to 2006 were excluded as height-for-age (HAZ) score standards set by World Health Organization (WHO) became effective only in 2006. Error bars correspond to 95% confidence intervals.

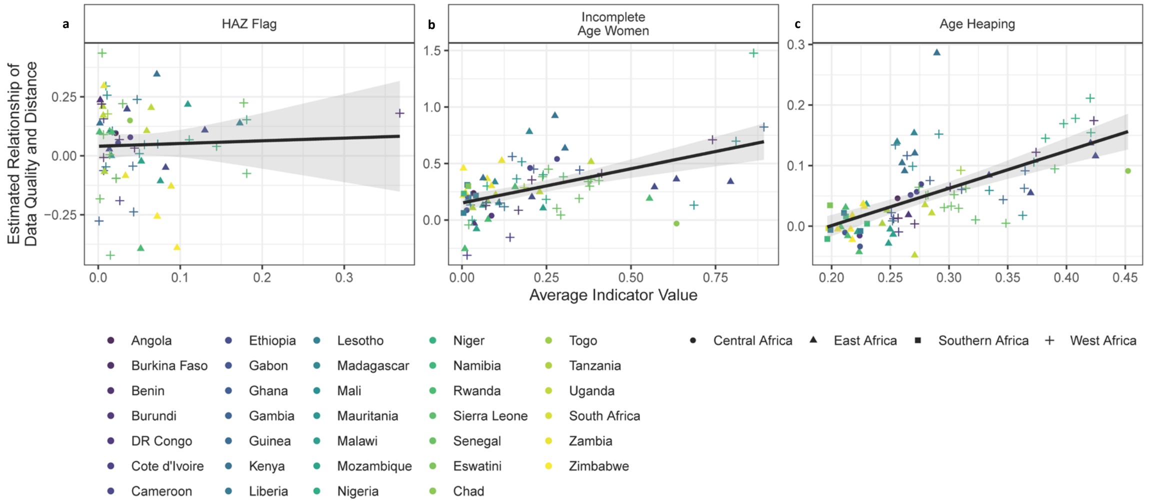

Supplementary Figure 12: Associations between estimated coefficients of distance to the closest Digital Number (DN) 15 nightlight pixels and the average value of data quality indicators. Coefficients obtained from binomial regressions of the data quality indicators on the distance measure and an intercept term. Shaded area corresponds to 95% confidence interval for predictions from the linear model.

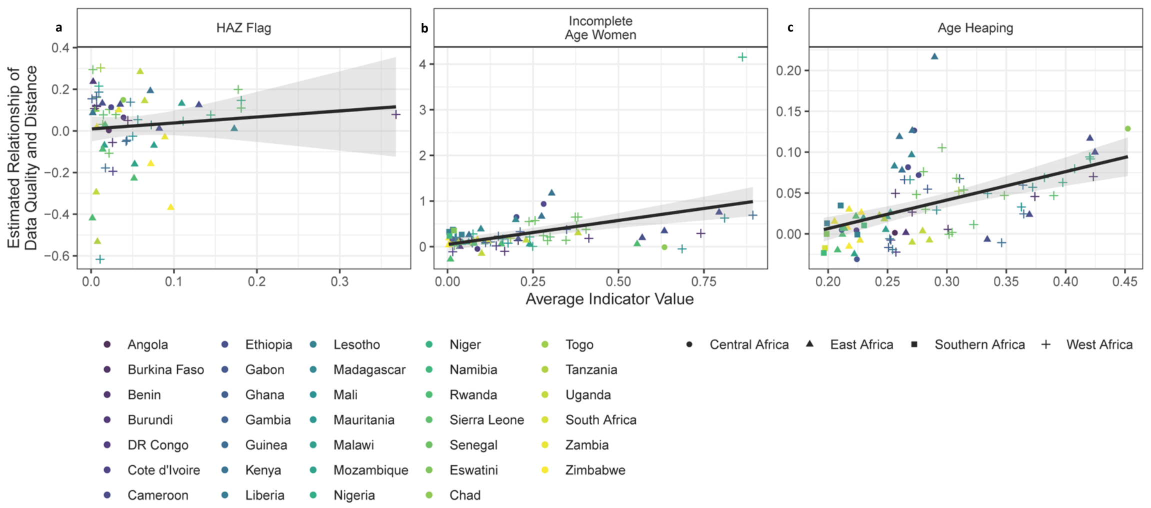

Supplementary Figure 13: Associations between estimated coefficients of distance to the closest settlement and the average value of data quality indicators. Coefficients obtained from binomial regressions of the data quality indicators on the distance measure and an intercept term. Shaded area corresponds to 95% confidence interval for predictions from the linear model.

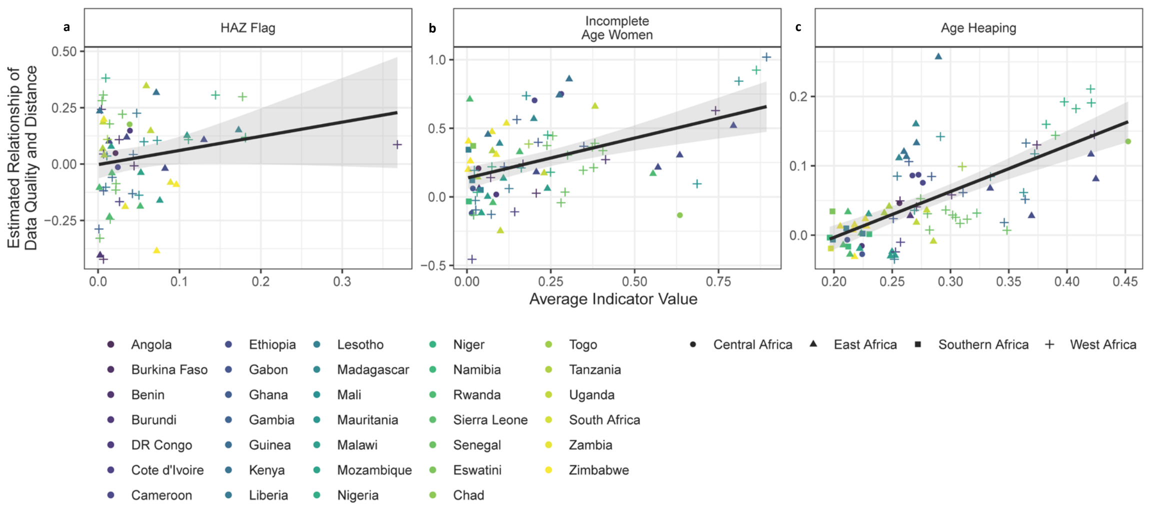

Supplementary Figure 14: Associations between estimated coefficients of distance to the closest urban agglomeration of at least 10,000 inhabitants and the average value of data quality indicators. Coefficients obtained from binomial regressions of the data quality indicators on the distance measure and an intercept term. Shaded area corresponds to 95% confidence interval for predictions from the linear model.

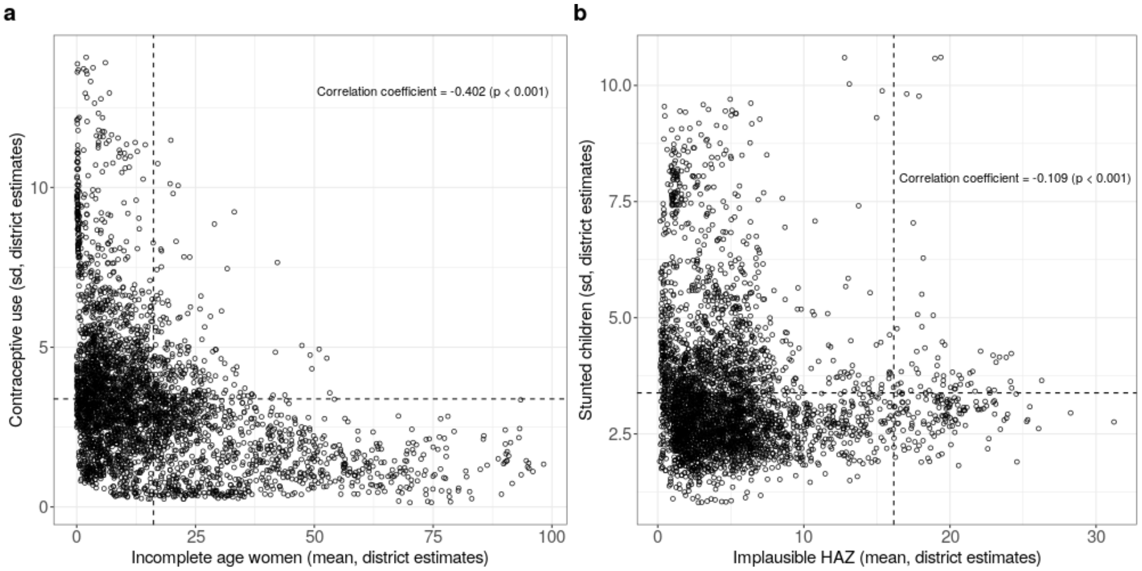

Supplementary Figure 15: Correlations between a) the sampling uncertainty of ’contraceptive use’ with ’incomplete age’ and b) of ’stunted children’ with ’flagged height-for-age (HAZ)'. Coefficients based on estimates at the district level.

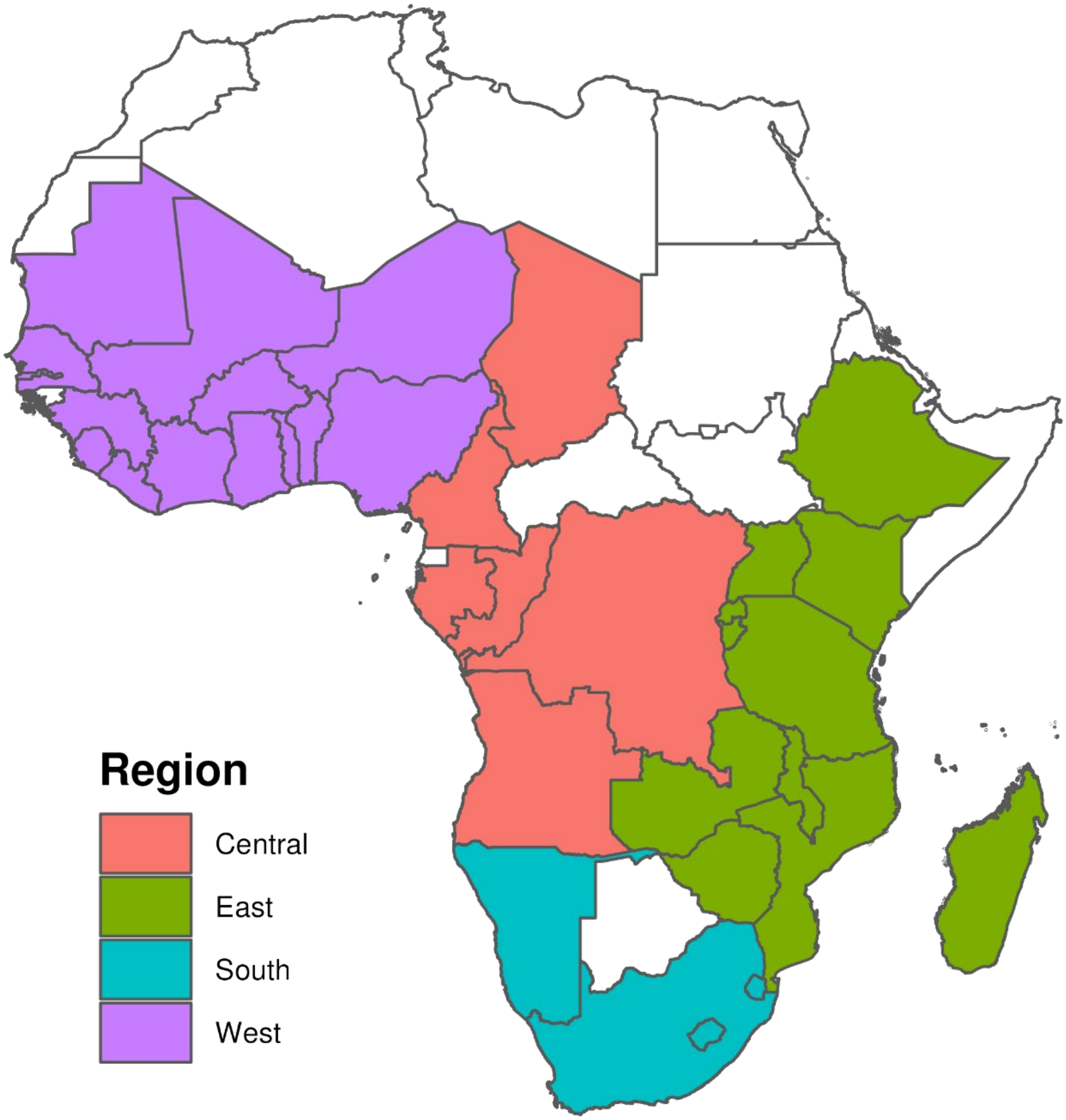

Supplementary Figure 16: Modelling regions in sub-Saharan Africa. Modelling regions were defined along the four Global Burden of Disease regions in sub-Saharan Africa: central, east, south and west. Countries in white were not included in the analysis**.**

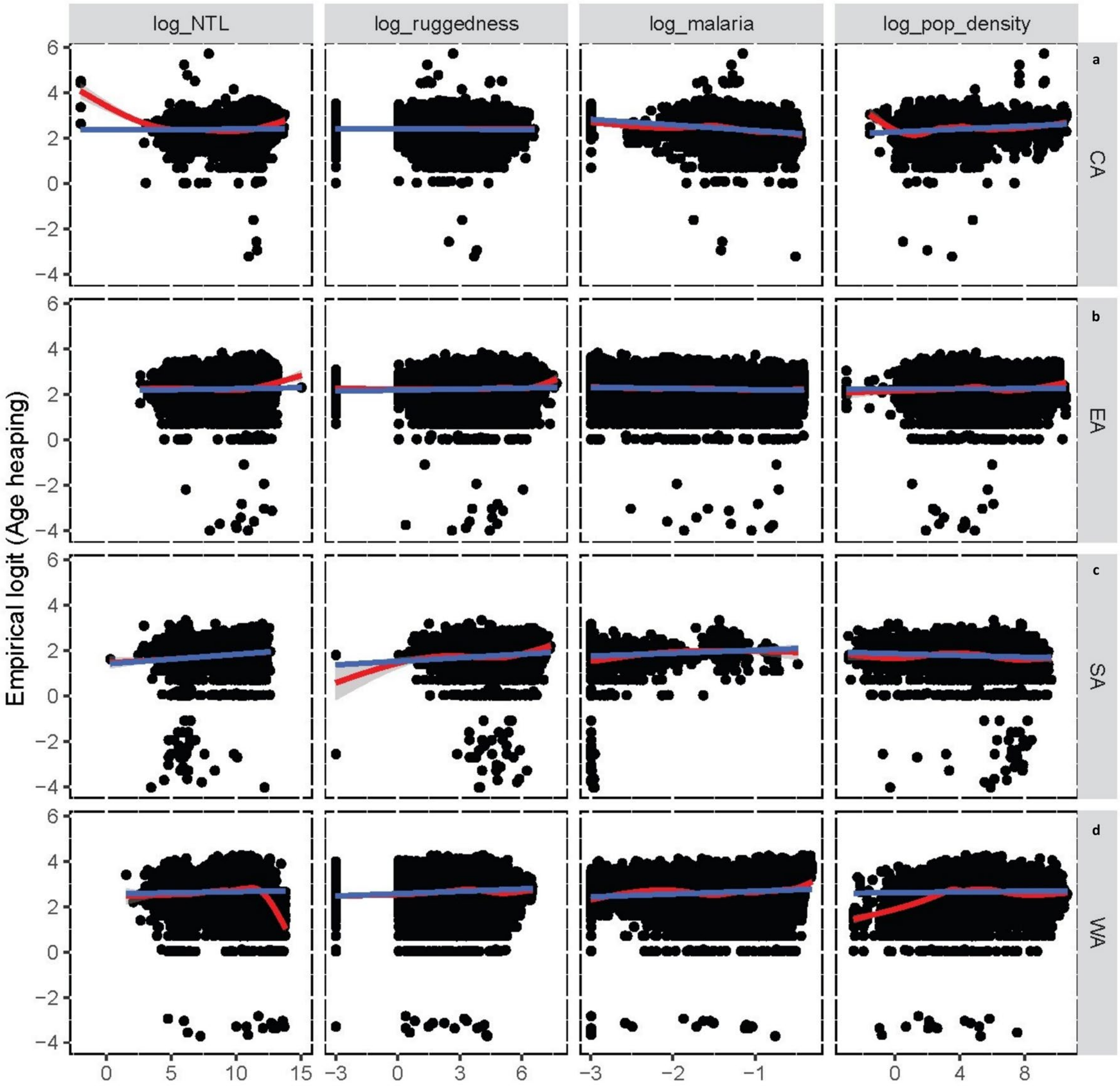

Supplementary Figure 17: Sample plots of the covariates against the empirical logit transform of age heaping across four African regions. The blue lines are simple least square fits to the data while the red lines and grey colored bands are generalized additive model (GAM) fits (with smooth functions represented using penalized regression splines) and corresponding uncertainty intervals. These plots generally show no evidence of nonlinearity between the covariates and age heaping.

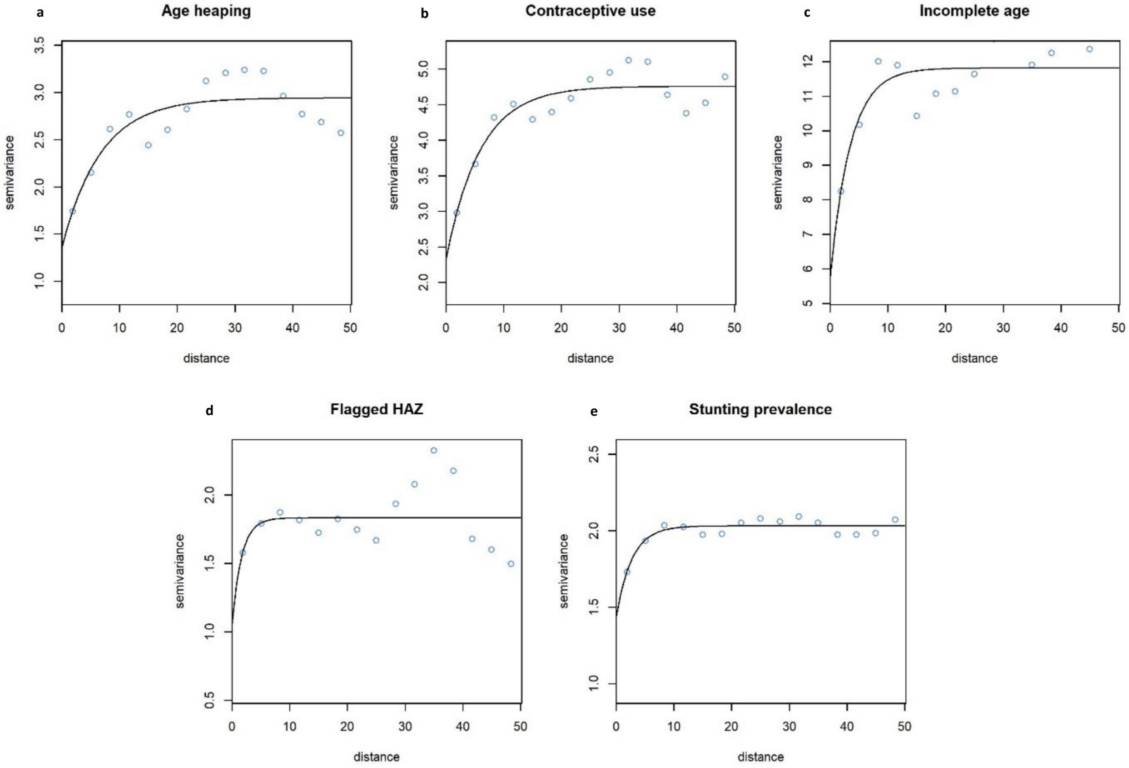

Supplementary Figure 18: Plots of variograms of residuals from non-spatial models fitted using each of the modelled indicators. These plots generally indicate the presence of significant residual spatial correlation, justifying the use of geostatistical models to predict these indicators.

Supplementary Tables

Covariate	Period	Dataset source	Reference / originally published in
Population density	Annual, 2000 – 2018	Gridded Population of the World (GPW), v4	Center For International Earth Science Information Network Columbia University. Gridded Population of the World, Version 4: Population Density. (2016)
Terrain ruggedness	Static	Diego Puga's website https://diegopuga.org/data/rugged/)	Nunn, N. & Puga, D. Ruggedness: The Blessing of Bad Geography in Africa. *Review of Economics and Statistics* **94** (2012)
Malaria incidence	Annual, 2000 – 2019	Malaria Atlas Project	Weiss, D. J. et al. Mapping the global prevalence, incidence, and mortality of Plasmodium falciparum, 2000–17: a spatial and temporal modelling study. *The Lancet* **394** (2019)
Nighttime light intensity	Annual, 1992 – 2018	Reprocessed dataset by Li et al. 2020	Li, X., Zhou, Y., Zhao, M. et al. A harmonized global nighttime light dataset 1992–2018. *Scientific Data* **7**, 168 (2020)
High-resolution data on settlements	2015	World Settlement Footprint 2015	Marconcini, M. et al. Outlining where humans live, the World Settlement Footprint 2015. *Scientific Data* **7** (2020)
Urban agglomerations	2015	Africapolis at www.africapolis.org	OECD/SWAC. Africapolis (database) (2020)

Supplementary Table 1: Sources for covariates and auxiliary data. OECD/SWAC stands for the Organisation for Economic Co-operation and Development / Sahel and West Africa Club.

Covariate	Min.	First quartile	Median	Mean	Third quartile	Max.	Std. Dev.
Nightlight	0.09	2455.06	30596.4	57029.23	72439.43	3345207	0.09
Malaria	0	0.07	0.19	0.22	0.35	0.68	0
Ruggedness	0	10.95	34.09	90.45	95.67	2173.26	0
Pop. density	0	41.75	144.14	1054.02	525.37	41127.57	0

Supplementary Table 2: Summary statistics for the covariates

Indicator	Correlation	RMSE	MAE	%Bias
**Central Africa**				
Age heaping	0.76	0.08	0.06	-0.08
Flagged HAZ	0.20	0.07	0.04	-1.11
Stunting prev.	0.58	0.16	0.13	1.59
Contraceptive use	0.77	0.11	0.08	0.19
Incomplete age	0.86	0.15	0.09	1.45
**East Africa**				
Age heaping	0.59	0.08	0.06	-0.03
Flagged HAZ	0.29	0.09	0.06	-2.43
Stunting prev.	0.49	0.18	0.15	2.28
Contraceptive use	0.68	0.13	0.11	-0.46
Incomplete age	0.68	0.19	0.13	-0.54
**Southern Africa**				
Age heaping	0.07	0.08	0.06	0.34
Flagged HAZ	0.22	0.12	0.07	1.50
Stunting prev.	0.29	0.24	0.20	2.97
Contraceptive use	0.40	0.17	0.13	-0.95
Incomplete age	0.36	0.04	0.02	0.18
**West Africa**				
Age heaping	0.60	0.10	0.07	0.12
Flagged HAZ	0.40	0.13	0.08	-1.20
Stunting prev.	0.48	0.17	0.13	0.89
Contraceptive use	0.61	0.11	0.08	0.01
Incomplete age	0.62	0.24	0.17	0.48

Supplementary Table 3: Results of k-fold cross-validation exercise to assess the predictive performance of the fitted models for each African region. HAZ stands for height-for-age.

Region	Posterior summary	Intercept	log(night light)*	log(ruggedness)*	log(malaria prev.)*	log(pop. dens.)*	Spatial range	Spatial variance	IID variance
Central Africa	Mean	-0.8498	0.0029	**-0.0170**	-0.0128	-0.0125	0.2747	0.2028	0.0201
	Std. dev.	0.2610	0.0038	0.0072	0.0160	0.0081	0.0662	0.0794	0.0029
	0.025	-1.3816	-0.0045	-0.0312	-0.0443	-0.0285	0.1778	0.0990	0.0148
	0.975	-0.3034	0.0103	-0.0029	0.0186	0.0034	0.4349	0.4051	0.0261
East Africa	Mean	-0.9201	0.0022	0.0004	-0.0030	**-0.0165**	0.0934	0.1225	0.0388
	Std. dev.	0.0756	0.0028	0.0047	0.0057	0.0061	0.0121	0.0228	0.0018
	0.025	-1.0673	-0.0033	-0.0089	-0.0143	-0.0284	0.0742	0.0872	0.0355
	0.975	-0.7670	0.0077	0.0097	0.0082	-0.0046	0.1213	0.1760	0.0424
Southern Africa	Mean	-1.3588	-0.0059	0.0101	0.0076	-0.0201	0.0390	0.0037	0.0033
	Std. dev.	0.0525	0.0110	0.0167	0.0082	0.0232	0.0339	0.0024	0.0026
	0.025	-1.4642	-0.0275	-0.0231	-0.0087	-0.0657	0.0066	0.0008	0.0002
	0.975	-1.2576	0.0157	0.0426	0.0234	0.0255	0.1309	0.0098	0.0097
West Africa	Mean	-0.6205	**0.0320**	-0.0065	**0.1289**	**-0.0545**	0.0415	0.1080	0.0815
	Std. dev.	0.0705	0.0039	0.0042	0.0111	0.0117	0.0143	0.0262	0.0020
	0.025	-0.7591	0.0244	-0.0148	0.1071	-0.0774	0.0170	0.0575	0.0775
	0.975	-0.4820	0.0397	0.0018	0.1506	-0.0315	0.0668	0.1562	0.0855

Supplementary Table 4: Parameter estimates for age heaping Parameter estimates shown in bold indicate significant associations. *Note that the covariates were standardized after the log-transformation to make their estimated coefficients comparable. Significant covariates are in bold font.

Region	Posterior summary	Intercept	log(night light)*	log(ruggedness)*	log(malaria prev.)*	log(pop. dens.)*	Spatial range	Spatial variance	IID variance
Central Africa	Mean	-3.8070	-0.0272	0.0620	0.0819	-0.0806	0.0535	0.4974	0.6594
	Std. dev.	0.1284	0.0216	0.0408	0.0793	0.0454	0.0100	0.1073	0.0765
	0.025	-4.0644	-0.0694	-0.0178	-0.0722	-0.1699	0.0370	0.3191	0.5215
	0.975	-3.5553	0.0152	0.1422	0.2391	0.0084	0.0760	0.7390	0.8215
East Africa	Mean	-3.7184	**-0.0532**	-0.0180	**0.2452**	**-0.1561**	0.0811	0.8687	1.1800
	Std. dev.	0.1911	0.0132	0.0232	0.0272	0.0291	0.0104	0.1455	0.0379
	0.025	-4.0916	-0.0790	-0.0635	0.1920	-0.2134	0.0628	0.6212	1.1057
	0.975	-3.3326	-0.0272	0.0276	0.2986	-0.0990	0.1036	1.1905	1.2543
Southern Africa	Mean	-4.7870	-0.0143	**0.4684**	-0.0139	0.2217	0.0419	0.4745	0.4778
	Std. dev.	0.5968	0.0815	0.1310	0.0383	0.2071	0.0265	0.3003	0.1400
	0.025	-6.0754	-0.1692	0.2111	-0.0938	-0.1778	0.0119	0.1206	0.2463
	0.975	-3.7243	0.1507	0.7258	0.0567	0.6351	0.1121	1.2599	0.7901
West Africa	Mean	-3.8714	**-0.0688**	0.0210	**1.1499**	**-0.2015**	0.0757	1.3654	1.9797
	Std. dev.	0.2778	0.0189	0.0200	0.0595	0.0563	0.0098	0.2492	0.0476
	0.025	-4.4059	-0.1059	-0.0183	1.0335	-0.3123	0.0587	0.9444	1.8895
	0.975	-3.3015	-0.0318	0.0603	1.2670	-0.0912	0.0970	1.9187	2.0762

Supplementary Table 5: Parameter estimates for flagged height-for-age (HAZ) Parameter estimates shown in bold indicate significant associations. *Note that the covariates were standardized after the log-transformation to make their estimated coefficients comparable.

Region	Posterior summary	Intercept	log(night light)*	log(ruggedness)*	log(malaria prev.)*	log(pop. dens.)*	Spatial range	Spatial variance	IID variance
Central Africa	Mean	-0.3662	**0.0905**	0.0114	**0.1062**	**-0.0459**	0.0920	0.1816	0.0970
	Std. dev.	0.1018	0.0082	0.0156	0.0324	0.0182	0.0167	0.0399	0.0115
	0.025	-0.5660	0.0744	-0.0192	0.0429	-0.0816	0.0653	0.1178	0.0758
	0.975	-0.1591	0.1067	0.0421	0.1699	-0.0102	0.1304	0.2739	0.1208
East Africa	Mean	-0.6118	**0.0718**	**0.0559**	**0.0467**	**-0.1183**	0.0373	0.1891	0.1575
	Std. dev.	0.0486	0.0058	0.0092	0.0108	0.0122	0.0033	0.0191	0.0065
	0.025	-0.7088	0.0604	0.0379	0.0255	-0.1423	0.0315	0.1552	0.1450
	0.975	-0.5173	0.0833	0.0739	0.0679	-0.0945	0.0443	0.2301	0.1705
Southern Africa	Mean	-0.6163	0.0250	**0.1350**	-0.0148	**-0.2391**	0.0859	0.1334	0.0821
	Std. dev.	0.2697	0.0346	0.0559	0.0248	0.0852	0.0664	0.1024	0.0327
	0.025	-1.2024	-0.0430	0.0248	-0.0658	-0.4064	0.0191	0.0267	0.0308
	0.975	-0.1177	0.0931	0.2443	0.0318	-0.0717	0.2654	0.4083	0.1564
West Africa	Mean	-0.7685	**0.1145**	-0.0013	**0.2380**	**-0.1359**	0.0478	0.1058	0.1971
	Std. dev.	0.0551	0.0071	0.0075	0.0196	0.0200	0.0060	0.0153	0.0080
	0.025	-0.8798	0.1006	-0.0160	0.1995	-0.1752	0.0377	0.0809	0.1837
	0.975	-0.6620	0.1285	0.0133	0.2765	-0.0967	0.0612	0.1407	0.2148

Supplementary Table 6: Parameter estimates for stunting prevalence ('stunted children'). Parameter estimates shown in bold indicate significant associations. *Note that the covariates were standardized after the log-transformation to make their estimated coefficients comparable

Region	Posterior summary	Intercept	log(night light)*	log(rugged ness)*	log(malaria prev.)*	log(pop. dens.)*	Spatial range	Spatial variance	IID variance
Central Africa	Mean	-2.9332	**-0.1003**	0.0259	-0.0628	**0.0798**	0.0982	1.8303	0.2707
	Std. dev.	0.3310	0.0104	0.0194	0.0389	0.0225	0.0131	0.3823	0.0145
	0.025	-3.6125	-0.1207	-0.0122	-0.1393	0.0357	0.0764	1.2266	0.2430
	0.975	-2.2950	-0.0800	0.0640	0.0135	0.1240	0.1277	2.7196	0.3000
East Africa	Mean	-1.7692	**-0.0421**	0.0033	**-0.1834**	**0.1611**	0.0532	1.2608	0.2370
	Std. dev.	0.1599	0.0058	0.0090	0.0107	0.0121	0.0040	0.1496	0.0055
	0.025	-2.0898	-0.0535	-0.0144	-0.2045	0.1373	0.0459	0.9998	0.2261
	0.975	-1.4588	-0.0308	0.0210	-0.1624	0.1849	0.0617	1.5856	0.2478
Southern Africa	Mean	-0.2644	**-0.0903**	-0.0474	0.0025	-0.0318	0.0370	0.1498	0.1735
	Std. dev.	0.1255	0.0185	0.0313	0.0131	0.0396	0.0098	0.0476	0.0152
	0.025	-0.5128	-0.1266	-0.1088	-0.0233	-0.1096	0.0219	0.0779	0.1452
	0.975	-0.0155	-0.0540	0.0142	0.0282	0.0457	0.0603	0.2633	0.2046
West Africa	Mean	-2.4839	**-0.1058**	0.0092	**-0.4446**	**0.1857**	0.0303	0.7628	0.3498
	Std. dev.	0.0957	0.0088	0.0091	0.0230	0.0272	0.0020	0.0684	0.0083
	0.025	-2.6735	-0.1230	-0.0087	-0.4897	0.1325	0.0268	0.6418	0.3338
	0.975	-2.2965	-0.0885	0.0270	-0.3995	0.2391	0.0346	0.9099	0.3664

Supplementary Table 7: Parameter estimates for contraceptive use. Parameter estimates shown in bold indicate significant associations. *Note that the covariates were standardized after the log-transformation to make their estimated coefficients comparable.

Region	Posterior summary	Intercept	log(night light)*	log(ruggedness)*	log(malaria prev.)*	log(pop. dens.)*	Spatial range	Spatial variance	IID variance
Central Africa	Mean	-2.3698	**0.0988**	-0.0721	**1.1177**	0.0249	0.0780	4.9233	1.5667
	Std. dev.	0.4537	0.0230	0.0405	0.0846	0.0522	0.0087	0.7861	0.0655
	0.025	-3.2554	0.0536	-0.1516	0.9521	-0.0778	0.0626	3.5741	1.4412
	0.975	-1.4573	0.1441	0.0073	1.2840	0.1272	0.0969	6.6545	1.6983
East Africa	Mean	-2.1101	**0.1799**	-0.0126	**0.4044**	**-0.4629**	0.0539	3.4505	2.2990
	Std. dev.	0.2683	0.0144	0.0220	0.0270	0.0296	0.0041	0.3694	0.0377
	0.025	-2.6369	0.1517	-0.0557	0.3513	-0.5211	0.0465	2.7804	2.2261
	0.975	-1.5793	0.2083	0.0306	0.4575	-0.4050	0.0625	4.2283	2.3742
Southern Africa	Mean	-7.1332	**0.2888**	**0.5695**	-0.0655	-0.0481	0.0543	3.1533	0.6974
	Std. dev.	0.9920	0.0889	0.1300	0.0904	0.2385	0.0210	1.8359	0.1235
	0.025	-9.3704	0.1179	0.3155	-0.2666	-0.5039	0.0255	0.9873	0.4829
	0.975	-5.4504	0.4669	0.8257	0.0872	0.4326	0.1069	7.9716	0.9663
West Africa	Mean	-1.1838	**0.2204**	0.0009	**1.2953**	**-0.6583**	0.0385	5.4111	3.7189
	Std. dev.	0.3033	0.0232	0.0230	0.0591	0.0696	0.0029	0.5042	0.0602
	0.025	-1.7778	0.1750	-0.0443	1.1794	-0.7953	0.0335	4.4942	3.6030
	0.975	-0.5827	0.2659	0.0461	1.4114	-0.5219	0.0447	6.4706	3.8393

Supplementary Table 8: Parameter estimates for incomplete age Parameter estimates shown in bold indicate significant associations. *Note that the covariates were standardized after the log-transformation to make their estimated coefficients comparable.

Country	DHS surveys 2006-2022	Year selected	Geographic Detail
Angola	2015	2015	GPS
Benin	2010/11, 2017/18	2017/18	GPS
Burkina Faso	2010/11, 2021	2021	GPS
Burundi	2016/17	2016/17	GPS
Cameroon	2011, 2018	2018	GPS
Chad	2014/15	2014/15	GPS
Congo	2011/12	2011/12	Admin 1 level
Congo Democratic Republic	2013/14	2013/14	GPS
Cote D'Ivoire	2011/12, 2021	2021	GPS
Eswatini	2006/07	2006/07	GPS
Ethiopia	2010/11, 2016	2016	GPS
Gabon	2012, 2019	2019	GPS
Ghana	2008, 2014	2014	GPS
Gambia	2019	2019	GPS
Guinea	2012, 2018	2018	GPS
Kenya	2008/09, 2014, 2022	2022	GPS
Lesotho	2009, 2014	2014	GPS
Liberia	2013, 2019	2019	GPS
Madagascar	2008/09, 2021	2021	GPS
Malawi	2010, 2015/16	2015	GPS
Mali	2006, 2012/13, 2018	2018	GPS
Mauritania	2020	2020	GPS
Mozambique	2011	2011	GPS
Namibia	2006/07, 2013	2013	GPS
Niger	2012	2012	GPS
Nigeria	2008, 2013, 2018	2018	GPS
Rwanda	2008, 2010, 2014/15, 2019	2019	GPS
Senegal	2005, 2010/11, 2012/13, 2014 - 2019	2019	GPS
Sierra Leone	2008, 2013, 2019	2019	GPS
South Africa	2017	2017	GPS
Tanzania	2015/16, 2022	2022	GPS
Togo	2013/14	2013/14	GPS
Uganda	2006, 2011, 2016	2016	GPS
Zambia	2007, 2013/14, 2018	2018	GPS
Zimbabwe	2010/11, 2015	2015	GPS

Supplementary Table 9: Completed Demographic and Health Survey (DHS) rounds and selected datasets

Country	Moran's I value	p-value
Angola	0.034	0.0015
Benin	0.159	1.3875×10^{-24}
Burkina Faso	0.122	2.0221×10^{-11}
Burundi	0.136	2.9954×10^{-09}
Cameroon	0.256	6.0592×10^{-109}
Chad	0.435	3.6210×10^{-284}
Congo	0.275	4.3574×10^{-19}
Congo, Democratic Republic	0.097	7.9149×10^{-15}
Côte d'Ivoire	0.036	0.0027
Eswatini	0.027	*0.3486*
Ethiopia	0.192	3.2519×10^{-59}
Gabon	0.025	*0.0508*
Gambia	0.092	0.0003
Ghana	0.186	1.1985×10^{-22}
Guinea	0.177	1.0743×10^{-21}
Kenya	0.225	0.0000
Lesotho	0.030	*0.1636*
Liberia	0.200	1.3930×10^{-16}
Madagascar	0.235	9.4873×10^{-46}
Malawi	0.099	5.1518×10^{-15}
Mali	0.190	7.7180×10^{-17}
Mauritania	0.054	4.7820×10^{-32}
Mozambique	0.046	0.0003
Namibia	-0.012	*0.4511*
Niger	0.291	8.7664×10^{-132}
Nigeria	0.311	0.0000
Rwanda	0.021	*0.1407*
Senegal	0.125	3.5894×10^{-07}
Sierra Leone	0.298	7.1635×10^{-40}
South Africa	-0.003	*0.9193*
Tanzania	0.100	4.5086×10^{-20}
Togo	0.196	9.5662×10^{-14}
Uganda	0.044	0.0136
Zambia	0.035	0.0358
Zimbabwe	0.021	*0.2132*

Supplementary Table 10: Spatial autocorrelation for 'age heaping' at the district level. Moran's I statistic at the district level, p-value. An index value of close to +1 indicates clustering of similar estimates of 'age heaping' at the district level. An index value of close to 0 indicates no autocorrelation (perfect spatial randomness). An index value of close to -1 indicates dispersion or clustering of dissimilar values. Corresponding two-side p-values are presented. Non-significant are p-values in *italics.* All statistical comparisons were performed using two-sided Moran's I tests; with unadjusted values used for testing significance of each spatial clustering coefficient.

Country	Moran's I	p-value
Angola	0.018	*0.0703*
Benin	0.016	*0.2654*
Burkina Faso	0.004	*0.7521*
Burundi	-0.007	*0.8312*
Cameroon	0.044	0.0001
Chad	0.058	9.4960×10^{-07}
Congo	0.680	1.1972×10^{-106}
Congo, Democratic Republic	0.028	0.0176
Côte d'Ivoire	0.021	*0.0726*
Eswatini	0.094	0.0025
Ethiopia	0.012	*0.2425*
Gabon	-0.009	*0.6578*
Gambia	-0.012	*0.7570*
Ghana	0.008	*0.5931*
Guinea	0.039	0.0262
Kenya	0.010	*0.0566*
Lesotho	0.039	*0.0805*
Liberia	-0.026	*0.3608*
Madagascar	0.024	*0.1180*
Malawi	0.012	*0.3164*
Mali	0.185	4.4465×10^{-16}
Mauritania	-0.005	0.*3898*
Mozambique	0.135	9.8968×10^{-26}
Namibia	0.034	0.0061
Niger	0.147	1.2941×10^{-35}
Nigeria	0.070	1.0451×10^{-40}
Rwanda	-0.009	*0.6348*
Senegal	0.020	*0.3307*
Sierra Leone	0.002	*0.8765*
South Africa	0.004	*0.6011*
Tanzania	0.001	*0.8261*
Togo	-0.022	*0.4765*
Uganda	0.001	*0.8976*
Zambia	0.053	0.0018
Zimbabwe	0.035	*0.0501*

Supplementary Table 11: Spatial autocorrelation for ‘flagged height-for-age (HAZ)’ at the district level. Moran’s I statistic at the district level, p-value. An index value of close to +1 indicates clustering of similar estimates of ‘flagged HAZ’ at the district level. An index value of close to 0 indicates no autocorrelation (perfect spatial randomness). An index value of close to -1 indicates dispersion or clustering of dissimilar values. Corresponding two-side p-values are presented. Non-significant are p-values in *italics*. All statistical comparisons were performed using two-sided Moran's I tests; with unadjusted values used for testing significance of each spatial clustering coefficient.

Country	Moran's I	p-value
Angola	0.064	4.4374×10^{-09}
Benin	0.247	3.4086×10^{-56}
Burkina Faso	0.311	1.4852×10^{-64}
Burundi	0.363	9.2861×10^{-56}
Cameroon	0.482	0.0000
Chad	0.248	1.7107×10^{-94}
Congo	0.620	3.3853×10^{-89}
Congo, Democratic Republic	0.104	6.1844×10^{-17}
Côte d'Ivoire	0.105	2.0437×10^{-17}
Eswatini	0.135	0.0000
Ethiopia	0.105	4.7178×10^{-19}
Gabon	0.008	*0.4456*
Gambia	0.398	4.3581×10^{-53}
Ghana	0.294	1.2267×10^{-53}
Guinea	0.206	1.4670×10^{-28}
Kenya	0.446	0.0000
Lesotho	-0.009	*0.7804*
Liberia	0.216	4.7601×10^{-19}
Madagascar	0.344	3.8757×10^{-96}
Malawi	0.286	4.9052×10^{-112}
Mali	0.601	1.1047×10^{-150}
Mauritania	0.143	4.1145×10^{-214}
Mozambique	0.167	5.8015×10^{-38}
Namibia	0.005	*0.6049*
Niger	0.294	8.1712×10^{-135}
Nigeria	0.107	8.0995×10^{-92}
Rwanda	0.419	8.4249×10^{-166}
Senegal	0.302	3.9520×10^{-33}
Sierra Leone	0.366	4.5844×10^{-59}
South Africa	0.007	*0.4358*
Tanzania	0.148	2.4984×10^{-41}
Togo	0.450	1.2976×10^{-64}
Uganda	0.525	2.4318×10^{-180}
Zambia	0.412	9.0136×10^{-122}
Zimbabwe	0.027	*0.1278*

Supplementary Table 12: Spatial autocorrelation for 'incomplete age' at the district level. Moran's I statistic at the district level, p-value. An index value of close to +1 indicates clustering of similar estimates of 'incomplete age' at the district level. An index value of close to 0 indicates no autocorrelation (perfect spatial randomness). An index value of close to -1 indicates dispersion or clustering of dissimilar values. Corresponding two-side p-values are presented. Non-significant are p-values in *italics*. All statistical comparisons were performed using two-sided Moran's I tests; with unadjusted values used for testing significance of each spatial clustering coefficient.

Supplementary References

1 Jerven M. Poor numbers. In: Poor Numbers. Cornell University Press, 2013.

2 Devarajan S. Africa's statistical tragedy. *Rev Income Wealth* 2013; **59**: S9–15.

3 Abay KA. Measurement errors in agricultural data and their implications on marginal returns to modern agricultural inputs. *Agric Econ* 2020; **51**: 323–41.

4 Carletto C, Savastano S, Zezza A. Fact or artifact: The impact of measurement errors on the farm size–productivity relationship. *J Dev Econ* 2013; **103**: 254–61.

5 Gollin D, Udry C. Heterogeneity, measurement error, and misallocation: Evidence from African agriculture. *J Polit Econ* 2021; **129**: 1–80.

6 Carletto C, Gourlay S, Murray S, Zezza A. Cheaper, faster, and more than good enough: is GPS the new gold standard in land area measurement? 2017: 235–65.

7 Desiere S, Jolliffe D. Land productivity and plot size: Is measurement error driving the inverse relationship? *J Dev Econ* 2018; **130**: 84–98.

8 Gibson J, Kim B. Non-classical measurement error in long-term retrospective recall surveys. *Oxf Bull Econ Stat* 2010; **72**: 687–95.

9 Corsi DJ, Perkins JM, Subramanian SV. Child anthropometry data quality from Demographic and Health Surveys, Multiple Indicator Cluster Surveys, and National Nutrition Surveys in the West Central Africa region: are we comparing apples and oranges? *Glob Health Action* 2017; **10**: 1328185.

10 Perumal N, Namaste S, Qamar H, Aimone A, Bassani DG, Roth DE. Anthropometric data quality assessment in multisurvey studies of child growth. *Am J Clin Nutr* 2020; **112**: 806S-815S.

11 Arthi V, Beegle K, De Weerdt J, Palacios-López A. Not your average job: Measuring farm labor in Tanzania. *J Dev Econ* 2018; **130**: 160–72.

12 Baliki G, Brück T, Ferguson NT, Kebede SW. Fragility exposure index: Concepts, measurement, and application. *Rev Dev Econ* 2022; **26**: 639–60.

13 Naudé W, McGillivray M, Rossouw S. Measuring the vulnerability of subnational regions in South Africa. In: Measuring Vulnerability in Developing Countries. Routledge, 2014: 67–94.

14 Birth registration. UNICEF DATA. https://data.unicef.org/topic/child-protection/birth-registration/ (accessed Aug 21, 2023).

15 Larsen AF, Headey D, Masters WA. Misreporting Month of Birth: Diagnosis and Implications for Research on Nutrition and Early Childhood in Developing Countries. *Demography* 2019; **56**: 707–28.

16 Fayehun O, Ajayi AI, Onuegbu C, Egerson D. Age heaping among adults in Nigeria: evidence from the Nigeria Demographic and Health Surveys 2003–2013. *J Biosoc Sci* 2020; **52**: 132–9.

17 Rosenzweig SC. Age is measured with systematic measurement error in developing country surveys: A diagnosis and analysis of consequences. *Res Polit* 2021; **8**: 20531680211044068.

18 Cummins J. On the Use and Misuse of Child Height-for-Age Z-score in the Demographic and Health Surveys. University of California at Riverside, Department of Economics, 2013 https://EconPapers.repec.org/RePEc:ucr:wpaper:201417.

19 Randall S, Coast E. The quality of demographic data on older Africans. *Demogr Res* 2016; **34**: 143–74.

20 Leroy JL, Frongillo EA. Perspective: What Does Stunting Really Mean? A Critical Review of the Evidence. *Adv Nutr* 2019; **10**: 196–204.

21 Nutrition and Food Safety. Recommendations for data collection, analysis and reporting on anthropometric indicators in children under 5 years old. 2019. https://apps.who.int/iris/handle/10665/324791 (accessed Aug 15, 2023).

22 Perez-Heydrich C, Warren J, Burgert C, Emch M. Guidelines on the Use of DHS GPS Data DHS Spatial Analysis. 2013. http://dhsprogram.com/pubs/pdf/SAR8/SAR8.pdf.
