# Supplementary material for: Subnational variations in the quality of household survey data in sub-Saharan Africa
Source: Nat Commun. 2025 Apr 22;16:3771. doi: 10.1038/s41467-025-58776-5 (PMC12015360; doi:10.1038/s41467-025-58776-5)
Supplement: Supplementary file 2 — Reporting Summary [file 41467_2025_58776_MOESM2_ESM.pdf]

Corresponding author(s): Valentin Seidler

Last updated by author(s): Feb 22, 2025

# Reporting Summary

Nature Portfolio wishes to improve the reproducibility of the work that we publish. This form provides structure for consistency and transparency in reporting. For further information on Nature Portfolio policies, see our Editorial Policies and the Editorial Policy Checklist.

## Statistics

For all statistical analyses, confirm that the following items are present in the figure legend, table legend, main text, or Methods section.

| n/a | Confirmed | |
|---|---|---|
| ☐ | ☒ | The exact sample size ($n$) for each experimental group/condition, given as a discrete number and unit of measurement |
| ☐ | ☒ | A statement on whether measurements were taken from distinct samples or whether the same sample was measured repeatedly |
| ☐ | ☒ | The statistical test(s) used AND whether they are one- or two-sided<br>*Only common tests should be described solely by name; describe more complex techniques in the Methods section.* |
| ☐ | ☒ | A description of all covariates tested |
| ☐ | ☒ | A description of any assumptions or corrections, such as tests of normality and adjustment for multiple comparisons |
| ☐ | ☒ | A full description of the statistical parameters including central tendency (e.g. means) or other basic estimates (e.g. regression coefficient) AND variation (e.g. standard deviation) or associated estimates of uncertainty (e.g. confidence intervals) |
| ☐ | ☒ | For null hypothesis testing, the test statistic (e.g. $F$, $t$, $r$) with confidence intervals, effect sizes, degrees of freedom and $P$ value noted<br>*Give P values as exact values whenever suitable.* |
| ☐ | ☒ | For Bayesian analysis, information on the choice of priors and Markov chain Monte Carlo settings |
| ☒ | ☐ | For hierarchical and complex designs, identification of the appropriate level for tests and full reporting of outcomes |
| ☐ | ☒ | Estimates of effect sizes (e.g. Cohen's $d$, Pearson's $r$), indicating how they were calculated |

*Our web collection on statistics for biologists contains articles on many of the points above.*

## Software and code

Policy information about availability of computer code

| | |
|---|---|
| Data collection | Data has been downloaded from the official DHS website and we provide detailed sources in the Supplementary Information |
| Data analysis | All data pre-processing has been conducted in the open source statistical programming language R in version 4.2.1. Statistical analysis has also been conducted using R 4.2.1 and geospatial modeling was primarily based on R package INLA (version 22.05.07). Replication codes are available online at https://doi.org/10.5281/zenodo.14892010. |

For manuscripts utilizing custom algorithms or software that are central to the research but not yet described in published literature, software must be made available to editors and reviewers. We strongly encourage code deposition in a community repository (e.g. GitHub). See the Nature Portfolio guidelines for submitting code & software for further information.

## Data

Policy information about availability of data

All manuscripts must include a data availability statement. This statement should provide the following information, where applicable:

- Accession codes, unique identifiers, or web links for publicly available datasets
- A description of any restrictions on data availability
- For clinical datasets or third party data, please ensure that the statement adheres to our policy

The findings of this study are supported by DHS datasets that are publicly available online at https://dhsprogram.com/. A detailed description of the selected DHS datasets and completed DHS rounds per country can be found in Supplementary Table 9. Other referenced data used for covariates are taken from freely available

gridded datasets. (1) The Gridded Population of the World, Version 4 (GPWv4): Population Count. https://doi.org/DOI:10.7927/H4X63JVC. (2) The Malaria Atlas Project (MAP) https://data.malariaatlas.org/. (3) Terrain ruggedness was extracted from a data set assembled and collected by Nathan Nunn and Diego Puga in 2012 https://diegopuga.org/data/rugged/. (4) The nighttime light emissions dataset was generated by Li, Zhou, Zhao & Zhao https://doi.org/10.6084/m9.figshare.9828827.v2. (5) Settlements in the study were delineated by using both the satellite-based high-resolution settlement data from Marconcini et al. https://doi.org/10.6084/m9.figshare.c.4712852 and the Africapolis dataset at https://africapolis.org/en/data. The primary generated in this study and the data to replicate all findings are available at https://data.worldpop.org/repo/prj/dhs/SSA/data_quality.zip.

## Human research participants

Policy information about studies involving human research participants and Sex and Gender in Research.

| | |
|---|---|
| Reporting on sex and gender | No research was conducted directly with human research participants. The study is based on third party survey data collected from various populations throughout 35 African countries. During data processing, sex indicators were assigned based on the corresponding variables in the raw survey data. The surveys are mainly focused on female participants, hence, a number of indicators (e.g., missing age information) is only sensible to conduct based on survey data collected from females. Indicators based on both genders have been computed whenever possible (e.g., age heaping and child nutrition indicators). |
| Population characteristics | The population characteristics of the original surveys underlying our study vary slightly across surveys and years, but are in almost all cases representative of national populations in a given country and year, whereas certain age restrictions apply to female and male participants (most of the time, females in reproductive age between 15 and 49 are surveyed, whereas for males, 15-64 is a common age sampling frame). |
| Recruitment | No recruitment took place during this study as it relies on third party survey data. |
| Ethics oversight | No research was conducted directly with human research participants. Therefore, no ethics approval was deemed necessary. |

Note that full information on the approval of the study protocol must also be provided in the manuscript.

# Field-specific reporting

Please select the one below that is the best fit for your research. If you are not sure, read the appropriate sections before making your selection.

☐ Life sciences ☒ Behavioural & social sciences ☐ Ecological, evolutionary & environmental sciences

For a reference copy of the document with all sections, see nature.com/documents/nr-reporting-summary-flat.pdf

# Behavioural & social sciences study design

All studies must disclose on these points even when the disclosure is negative.

| | |
|---|---|
| Study description | We collect observations on data quality issues in a large number of household surveys in sub-Saharan Africa. We use geostatistical tools to describe a non-random spatial distribution of data quality and discuss potential underlying channels. |
| Research sample | Geocoded raw data from the DHS website permit a geostatistical analysis of the spatial distribution of data quality. The population characteristics of the original surveys underlying our study vary slightly across surveys and years, but are in almost all cases representative of national populations in a given country and year, whereas certain age restrictions apply to female and male participants (most of the time, females in reproductive age between 15 and 49 are surveyed, whereas for males, 15-64 is a common age sampling frame). |
| Sampling strategy | No specific sampling strategy was applied during this observational study as it is based on third party survey data. |
| Data collection | All raw data was downloaded directly from the DHS website and we provide detailed information on the survey sources in the Supplementary material. |
| Timing | Data collection started in December 2020 and ended in March 2024. |
| Data exclusions | We collected all DHS survey data available for African countries where geocoded survey data was available. For the raw microdata, the exclusion criteria superimposed by the DHS apply and include for instance separate codes for biologically implausible values. |
| Non-participation | As this is an observational study and no direct recruitment of participants took place, no non-participation issues are reported. |
| Randomization | The data in this study predominantly is based on third party survey data, which is usually based on randomized survey designs. Apart from that, being an observational study focused on mapping, no randomization took place. |

# Reporting for specific materials, systems and methods

We require information from authors about some types of materials, experimental systems and methods used in many studies. Here, indicate whether each material, system or method listed is relevant to your study. If you are not sure if a list item applies to your research, read the appropriate section before selecting a response.

## Materials & experimental systems

| n/a | Involved in the study |
|---|---|
| ☒ | ☐ Antibodies |
| ☒ | ☐ Eukaryotic cell lines |
| ☒ | ☐ Palaeontology and archaeology |
| ☒ | ☐ Animals and other organisms |
| ☒ | ☐ Clinical data |
| ☒ | ☐ Dual use research of concern |

## Methods

| n/a | Involved in the study |
|---|---|
| ☒ | ☐ ChIP-seq |
| ☒ | ☐ Flow cytometry |
| ☒ | ☐ MRI-based neuroimaging |
